# The influence of existing piles on station settlement during the construction of a tunnel undercrossing under existing stations

**DOI:** 10.1038/s41598-024-63921-z

**Published:** 2024-06-18

**Authors:** Baolin Ma, Shengzhi Wu, Qihui Chen, Erbin Liang, Xiudong Li

**Affiliations:** 1 School of Civil Engineering, Shandong Jianzhu University, Jinan, 250101 Shandong China; 2 China Railway 14 Bureau Group Co, Ltd., Jinan, 250000 China

**Keywords:** Tunnel, Undercrossing construction, Subway station, Existing piles, Sedimentation, Engineering, Civil engineering

## Abstract

In the construction of tunnels under existing stations, it is necessary to control their settlement. When there is a pile foundation in the existing station, the pile cutting has a significant impact on the settlement of the existing station. To determine the influence of existing piles on the settlement of subway stations, a reasonable pile-cutting time is proposed. Based on the Chengdu Metro Line 9 underpassing the existing Line 1 hatchery station, the settlement law of the tunnel underpassing the existing Line 1 station is analysed via a numerical simulation. Furthermore, the deformation and stress characteristics of the existing piles, pipe roofs, and tunnel linings and the supporting effect on the existing station are discussed. It is concluded that the cutting of existing piles causes a change in the tunnel bearing system, thus resulting in a certain deformation of the station. The influence of different pile cutting times on the settlement of the existing station is then analysed, and it is clarified that the tunnel support stiffness is significantly enhanced after the construction of the secondary lining. At this time, the settlement of the existing pile station is significantly reduced. Finally, through a field investigation, the effect of surface grouting, pipe shed, and multilayer lining on the settlement control of the existing station while the existing pile foundation exists is determined. This research can provide a reference for the settlement control and foundation underpinning of existing stations at ultra-small distances in underground excavation tunnels.

## Introduction

In recent years, subway construction in China has developed rapidly, and many of its cities have built subway operation networks. As subway lines form an operational network, the number of subway transfer stations has also increased. The construction process for subway transfer stations is complicated, and the underpass is constructed during the construction process. When a new tunnel passes through an existing station, the settlement control of the existing station is key to the operation of the existing station^1^.

With the continuous development of urban subway, shield tunnel crossing existing supporting piles has become an unavoidable engineering problem. Therefore, controlling the settlement of the existing station during the tunnel construction and ensuring the stability of the existing pile foundation are the common goals in engineering practice^2^. Scholars^3–5^ have studied the vertical displacement of the pile body through formula derivation and a series of centrifugal experiments and found that the settlement of the pile top increased by a greater extent than the surface settlement during the tunnel advancement^6^. Boonsiri and Zhang et al.^7–10^ found that pile groups affect surface movement. Zhang et al.^11^ proved the feasibility of a pile foundation underpinning construction scheme when a shield tunnel passes through existing piles, studied the comprehensive influence of shield tunnelling and the old pile cutting process on underpinning piles^12^, and proposed a pile foundation underpinning technology for an oblique tunnel crossing a pile group foundation^13^. Wang and Zhao^14–17^ proposed a series of strata reinforcement measures to significantly improve the deformation of existing structures and proposed a new grouting material for the pre-reinforcement of shield tunnels adjacent to existing pile foundations^18^. Wu^19^ and Wang^14^ analysed the mechanical properties of the pile when the shield tunnel passes under the bridge pile through numerical simulation, and evaluated the reliability of the reinforcement measures. Basile and Loganathan et al.^20–23^ evaluated the influence of tunnel excavation, tunnel depth, and soil inhomogeneity on existing pile foundations and provided reasonable pile-response predictions for many practical situations^24^. Chen^25^ and Wang^26^ studied the influence of different construction sequences on existing piles, and proposed a new construction method, i.e., the tunnel-column method^27^. Khabbaz^28^ and Wang^29^ analysed the tunnel–rock–pile, pile–shield machine, and tunnel boring machine–soil–pile interactions and the interaction force was calculated by model^30^. Zheng et al.^31^ studied the influence of shield tunnel on pile buildings. They got results that the settlement of the buildings was significantly affected by the position of the tunnel relative to the pile foundation. Ong et al.^32–35^ conducted centrifuge model tests to study the effects of soil movement induced by excavation on piles in clay, as well as the behavior of single piles and pile groups affected by soil movement in clay. The existing research demonstrates the influence of a new tunnel crossing the existing pile on the existing structure and proposes more settlement control methods for the existing station. Pile foundations were set up under these stations to control the long-term settlement of existing stations comprising weak strata. When a tunnel passes through a subway station, engineering piles must be removed. At present, relatively few studies have been conducted on the influence of existing engineering pile removal methods on station settlement.

Based on the Chengdu Metro Line 9 underpassing the existing Line 1 hatchery station, the settlement law of the tunnel underpassing the existing Line 1 station was analysed via a numerical simulation, and the deformation and stress characteristics of the existing piles, pipe roofs, and tunnel linings were discussed. The supporting effect of the existing station was determined, the influence of different pile cutting times on the settlement of the existing station was studied, and the control effect of the surface grouting, pipe roof, and multi-layer lining on the settlement of the existing station when there was an existing pile foundation was investigated on-site. This research can provide a reference for the settlement control and foundation underpinning of existing stations at ultra-small distances in underground excavation tunnels.

## Project overview

### Engineering location

The Incubation Park Station is located at the intersection of Jincheng Avenue, Jiaozi South Road, and Tianfu Avenue in the Chengdu High-tech Zone. The station is located partially under the Ladfang Building. It was opened on September 27, 2010. This is a transfer station for Chengdu Metro Lines 1 and 9. Among them, the station of Line 1 comprises a rare interval-side platform, which is similar to an island side platform. Incubation Park Station is the 16th station of the Chengdu Rail Transit Line 1. The total length of the station is 428.1 m, and the depth of the foundation pit of the standard section is approximately 14.6 m. The station has is a double-span single-column structure with three passenger entrances and exits, a barrier-free elevator, and two groups of wind pavilions.

**Figure 1:**
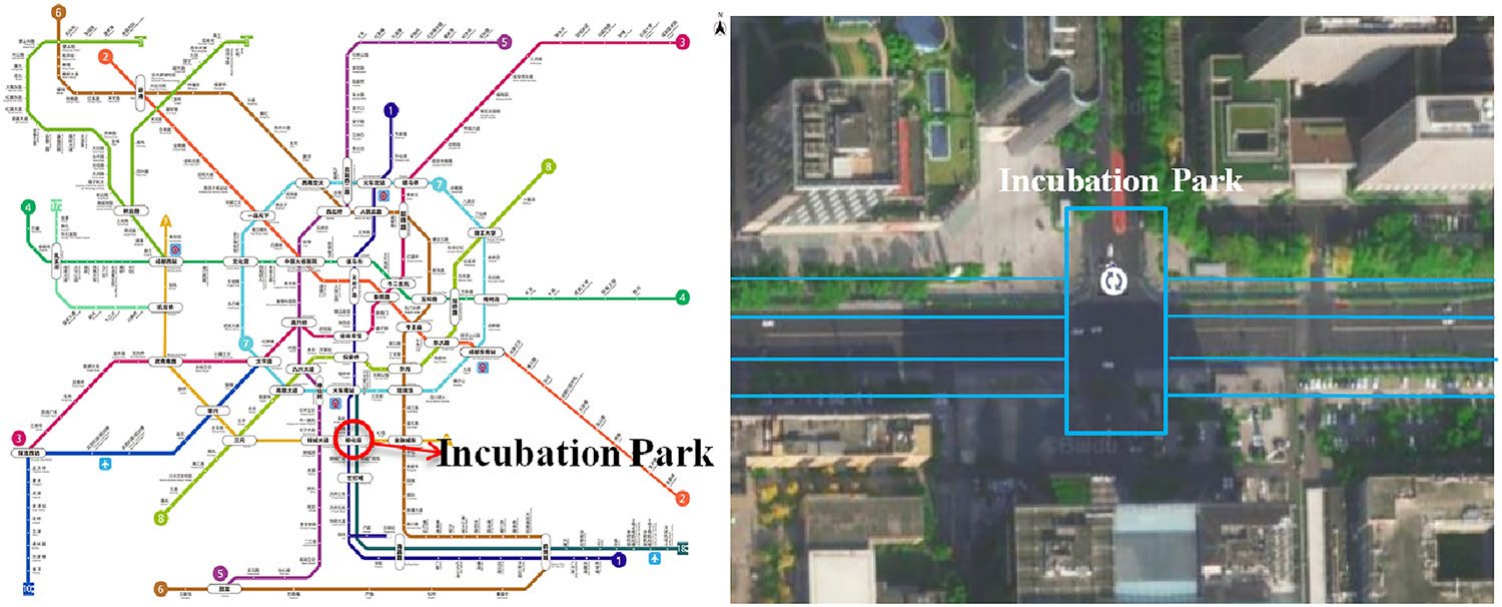
Plane location map of incubation park station. The map was created in AMap 13.13.2.2026 (https://wap.amap.com/).

The excavation section of the newly built tunnel is located below Jincheng Avenue and arranged in an east–west direction along Jincheng Avenue. It crosses under the existing station of Line 1 and transfers to the cross node of the station of Line 1. The vault of the new tunnel is only 2.3–2.6 m away from the station floor of Line 1, and there are six pile foundations under the station floor of Line 1, which extend into the underground excavation interval, as shown in Fig. 1.

### Engineering geology

**Figure 2:**
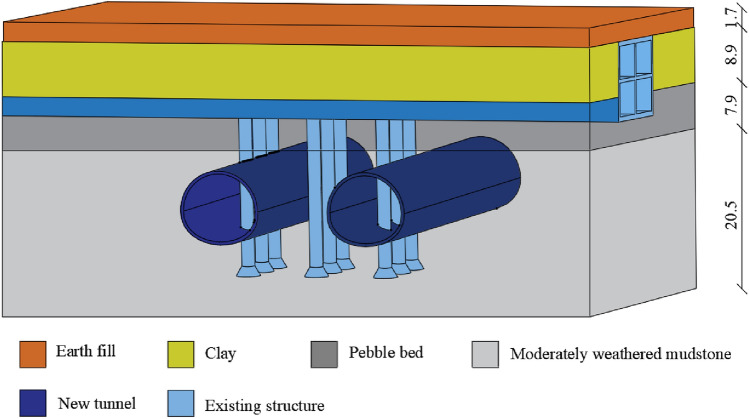
Geological map of hatchery station.

The soil layer where the tunnel is located includes soil, clay, pebble layer and moderately weathered mudstone from top to bottom, as shown in Fig. 2. The first layer is the filling soil layer, and the average thickness of the fill material is 1.7 m. The lower part of the filling layer is composed of clay soil, mainly comprising clay, with more silt in the local area. The average layer thickness is 8.9 m, and the depth of the top layer is 1.7 m. Beneath the clay layer is a pebble layer, with a pebble content of approximately 60–85% and particle size of 2–10 cm; the pebble colour is fresh with no weathering observed, the average layer thickness is 7.9 m, and the depth of the top layer is 10.6 m. Below the pebble layer is a moderately weathered mudstone composed mainly of clay minerals and an argillaceous structure. The rock mass is weathered into fragments, and local weathering has resulted in semi-rock and semi-soil. The core is easily softened in water. The buried depth of the top is 18.5 m. Groundwater is not observed in the strata of the station construction area.

### Tunnel excavation and support

**Figure 3:**
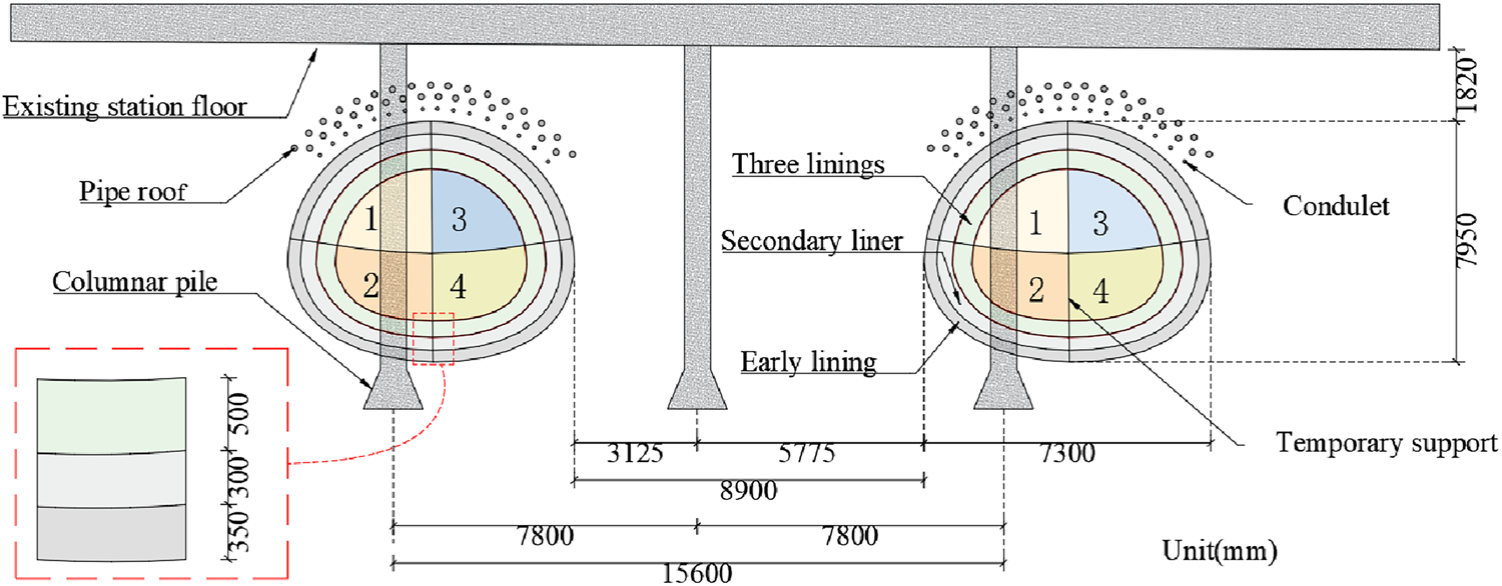
Schematic of cross-section excavation section of tunnel.

The mined tunnel is constructed using the cross diaphragm (CRD) method, the excavation sequence is as follows: (1) Excavation of the upper steps in the left pilot tunnel; (2) Excavation of the lower steps in the left pilot tunnel; (3) Excavation of the upper steps in the right pilot tunnel; (4) Excavation of the lower steps in the right pilot tunnel, and the partial excavation is presented in Fig. 3. The longitudinal distance between the left and right sides of the same section is 15m, and the longitudinal distance between the upper and lower parts is 3–5 m. Six supporting piles under the station floor of Line 1 are extended into the excavation section, and the excavation sections of the left and right lines pass through three piles, the parameters of the existing piles are listed in Table 1. After the secondary lining is poured and the expected strength is reached, the temporary support is removed, the pile is removed, and a third lining is constructed. Part of the supporting pile under the floor of the existing Line 1 station must be broken by the static force in the tunnel to avoid impacting the normal operation of Line 1.

**Table 1:** Existing pile parameters.

Type of pile	Pile diameter	Material	Pile length
End-bearing pile	1500 mm	C35 concrete, HPB300 and HRB400 grade rebar	12 m

The tunnel has a horseshoe-shaped cross-section with a composite lining. The excavation height is 7.95 m and the width is 7.3 m. The combined support type of shotcrete + steel mesh + steel arch + anchor rod + locking anchor rod is adopted, supplemented by advanced support measures such as advanced large pipe sheds, advanced small pipe grouting, and tunnel face shotcrete closure. The new tunnel is equipped with three linings. The initial support consisted of shotcrete, a steel mesh, a steel arch, and a self-drilling locking anchor. The initial support is 35-cm thick and the temporary support is 27-cm thick. The secondary and third linings comprise C35 reinforced concrete; the impermeability grade is P10, and the thicknesses of the second and third linings are 30 and 50 cm, respectively.

To reduce the disturbance to the soil around the station during the excavation of existing line 1, the soil between the bottom plate of the existing line 1 station and the vault of the new tunnel is grouted and reinforced. To ensure that the grouting does not affect the existing line structure, the grouting range is selected on both sides of the existing line 1 station, 2 m away from the existing line 1 station, and 0.5 m away from the outer edge of the retaining pile of line 9. The grouting construction has a plastic sleeve valve pipe grouting with a grouting depth of 16.9 m. The grouting slurry is ordinary cement slurry, the water–cement ratio of the cement slurry is 0.8:1–1:1, and the grouting pressure is 0.2–0.4 MPa.

To improve the stability of the middle soil between the existing station and underground tunnel, the middle soil is reinforced using the pipe curtain + small pipe method, and the position relationship is presented in Fig. 3. The pipe curtain is evenly arranged within 120° of the vault in the new tunnel. A seamless steel pipe with wall thickness of 6.0 mm is adopted, and the circumferential spacing is 0.35 cm. A Φ42 advanced small pipe is set under the pipe shed, with a wall thickness of 3.5 mm, a single root length of 1.5 m, and an extrapolation angle of 10°. After completion of the pipe shed and grouting construction, under the protection of comprehensive safeguard measures, each part is excavated according to the design method.

## Numerical simulation of settlement law of tunnel undercrossing existing station

### Numerical model

**Figure 4:**
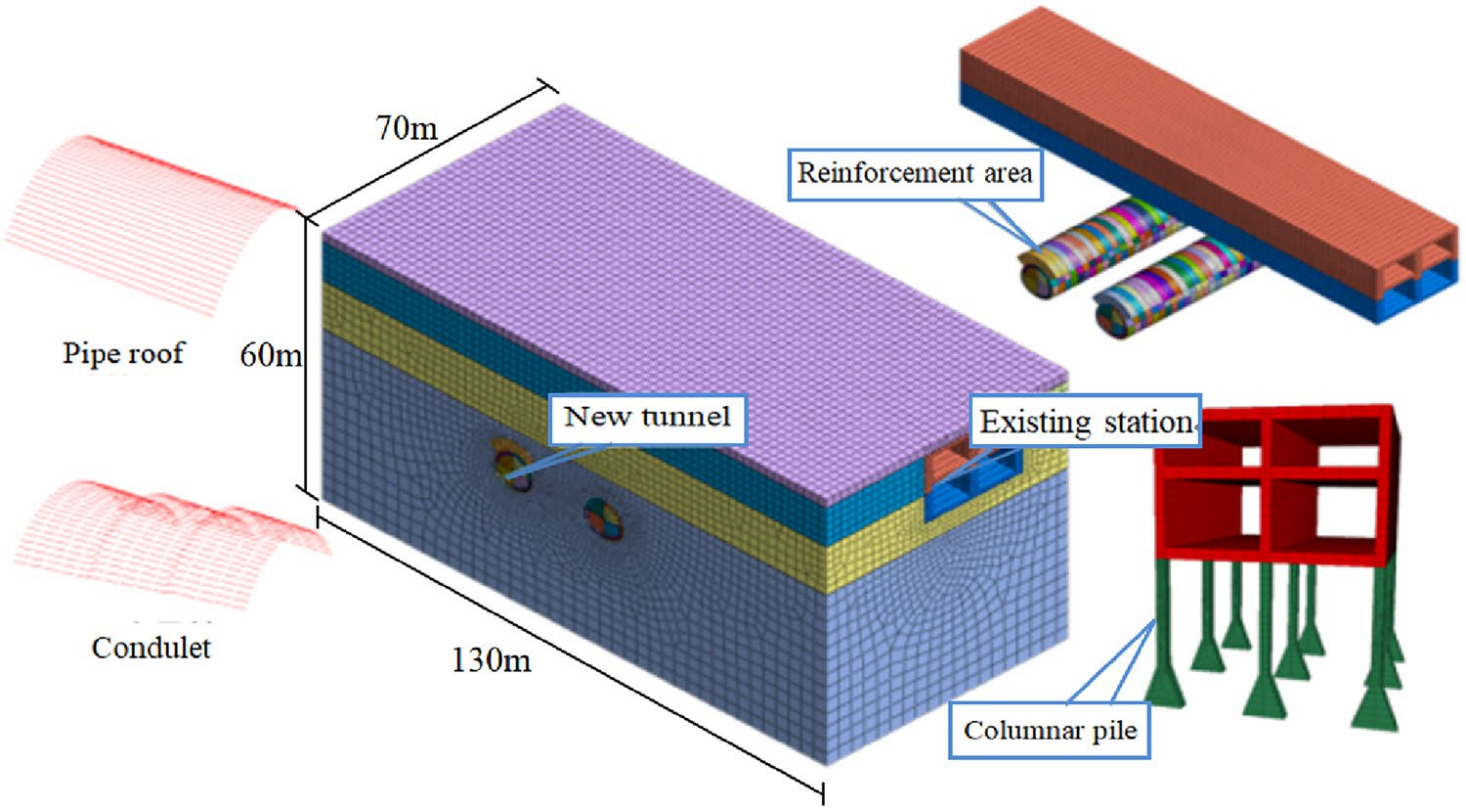
Numerical model diagram.

To determine the settlement law of the existing station during the tunnel construction, a three-dimensional finite element model is established using Midas GTS/NX. The model dimensions are 130 × 70 × 60 m. The Drucker–Prager constitutive model and beam element elastic constitutive model are adopted for the soil and the pipe roof, respectively, and the lining structure comprises a solid element. In the simulation, only the supporting piles are simulated for the lower pile foundation of the existing structure. The boundary conditions of the model comprise a fixed-end constraint at the bottom and free-hinged constraint in the Z-direction at the side boundary. The calculation model is illustrated in Fig. 4. The calculated parameters are listed in Table 2.

**Table 2:** Numerical simulation parameters.

Design conditions	Natural density (g/cm^3^)	Cohesion (kPa)	Internal friction angle (°)	Elastic modulus (GPa)	Poisson ratio	SPT(N/10cm)	Range
Station structure	2.5	–	–	31.5	0.2	–	–
Existing pile	2.5	–	–	30	0.2	–	–
C25 concrete	2.3	–	–	28	0.2	–	–
C35 concrete	2.5	–	–	31.5	0.2	–	–
Pipe roof	3.3	–	–	93	0.22	–	–
Surrounding rock of reinforced area	2.25	220	42	0.6	0.35	–	–
Earth fill	1.8	80	28	0.02	0.45	–	1.7m
Clay	2.0	180	30	0.1	0.4	2.44	8.9m
Pebble bed	2.15	120	36	0.4	0.38	9.49	7.9m
Moderately weathered mudstone	2.35	600	45	0.8	0.32	18.96	41.5m

To facilitate the study of the influence of different construction processes on the existing station, the construction step of first excavating the left-line tunnel is adopted in this model and the right-line tunnel is then excavated after the completion of the left-line tunnel construction. The specific construction steps include the left tunnel upper-left excavation, left tunnel lower-left excavation, left tunnel upper-right excavation, left tunnel lower-right excavation, left tunnel second-lining construction, left tunnel temporary support removal, left tunnel static pile removal, left tunnel third lining construction, and right tunnel excavation. The excavation steps for the right tunnel are identical to those of the left tunnel.

### Settlement law of existing structure

**Figure 5:**
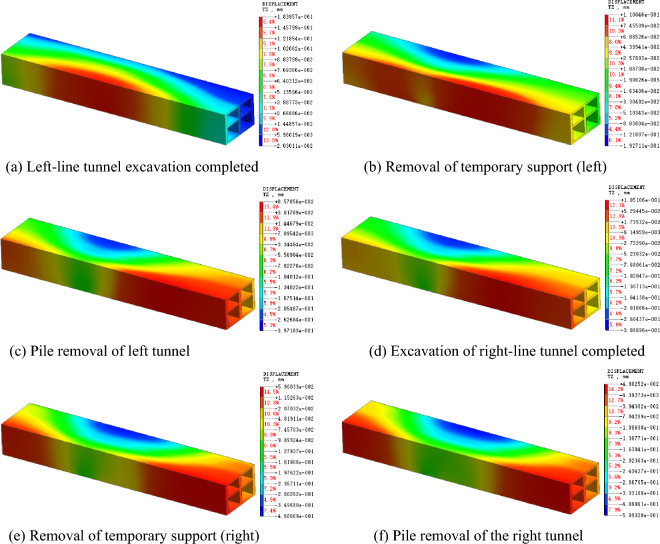
Settlement cloud diagram of existing station.

**Figure 6:**
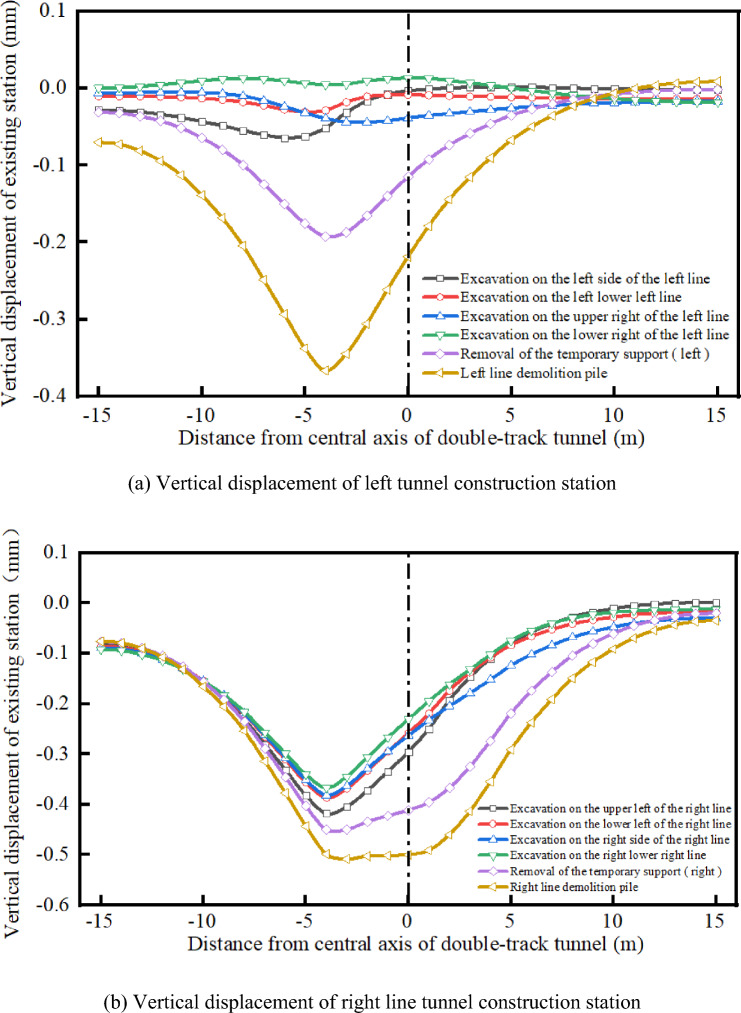
Settlement curve of existing station.

Figures 5 and 6 present the settlement cloud map and settlement curve of the station at different construction stages. The calculation results show that the settlement of the existing station above the tunnel is the greatest, and the settlements at both ends is small. When the left-line tunnel is constructed, the maximum settlement of the existing station is directly above the left-line tunnel and is 0.37 mm. When the construction of the right-line tunnel began, the settlement of the existing station began to move to the right, the settlement peak appeared again directly above the right line tunnel, and the final settlement was 0.49 mm directly above the right line tunnel. The tunnel had little influence on the existing station during excavation. When the left and right upper benches are excavated, the existing station is slightly settled, and is slightly jacked up when the left and right lower benches are excavated. After completion of the overall construction, the maximum settlement of the existing station is 0.51 mm.

**Figure 7:**
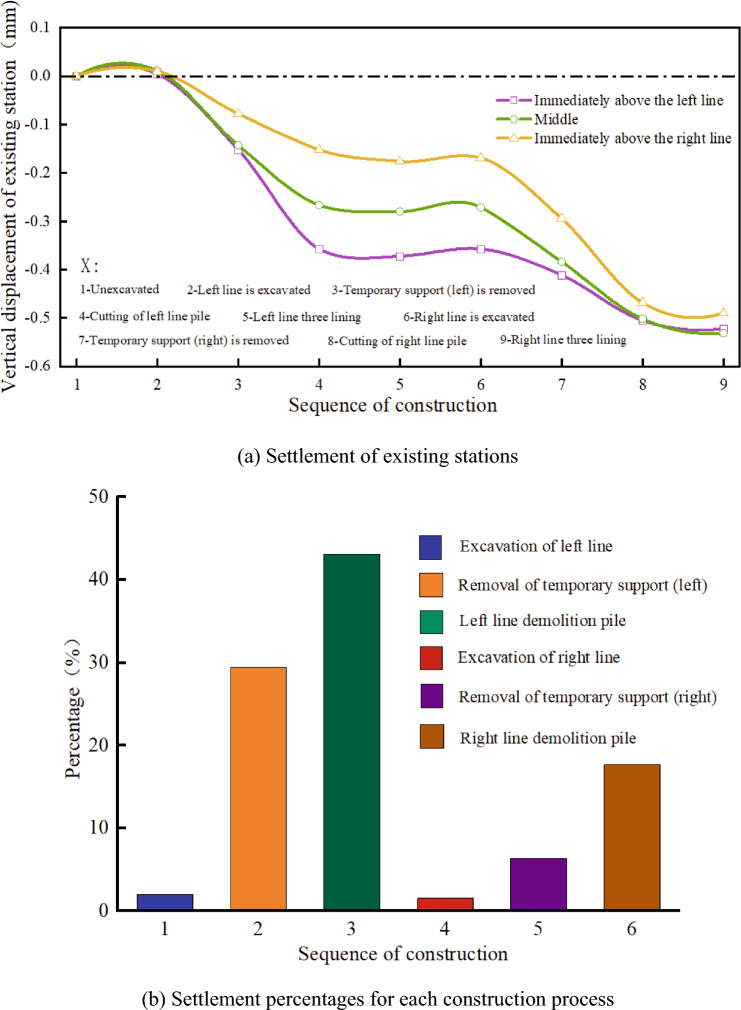
Settlement diagram of each construction stage.

Figure 7 presents the settlement characteristics of the existing stations at different construction stages. The diagram shows that the settlement of the existing station changes differently at different construction stages during the entire excavation process. After the excavation, the vertical displacement of the existing station is almost zero. When the temporary support is removed, the settlement of the existing station increases by 0.15 mm, which accounts for 29.41% of the total settlement. When the static pile is removed, the settlement at the existing station reaches its maximum value. At this time, the settlement of the existing station is 0.37 mm, which is an increase of 0.22 mm and accounts for 43.14% of the total settlement. After the construction of the left-line tunnel is completed, the excavation stage of the right-line tunnel has little effect on the left-line tunnel. When the temporary support is removed from the right-line tunnel, the settlement of the existing station increases again. In the pile removal stage of the right-line tunnel, the settlement of the existing station increases by 0.09 mm, which accounts for 17.65% of the total settlement.

It can be observed that, in the tunnel excavation process, the settlement of the station in the static pile removal stage accounts for the largest proportion of settlement, with the settlement of 0.37 mm and increment of 0.22 mm, which account for 60.79% of the total settlement at the existing stations. Therefore, the static pile removal stage is the control stage in the construction process.

### Deformation and stress law of existing pile

**Figure 8:**
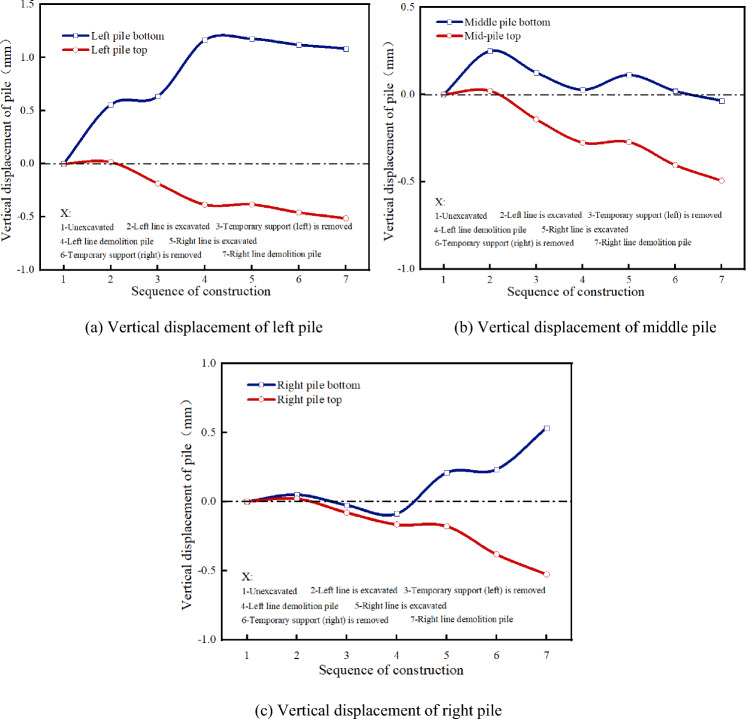
Vertical displacement of supporting piles.

**Figure 9:**
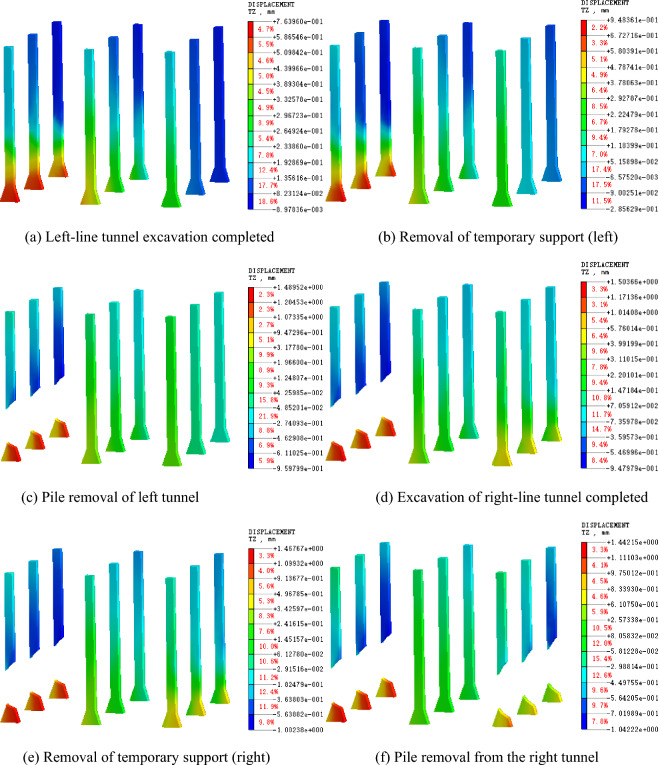
Displacement cloud diagram of supporting pile.

The existing piles have a better supporting effect on stations before demolition. Figures 8 and 9 present the deformation characteristics of the existing piles. After the excavation of the left tunnel, the soil at the bottom of the tunnel increases, which results in an overall upward movement of the left pile. However, owing to the influence of the existing station, the upward movement of the pile top is negligible. After the static demolition of the left tunnel, the settlement of the upper part of the left pile body increases significantly, and the lower part of the truncated pile body moves up owing to the upwelling of the soil at the bottom of the tunnel excavation.

The middle pile moves upward as a whole during the excavation stage of the left tunnel, and the pile top is slightly affected by the existing station. With the removal of the temporary support and static pile in the left tunnel, the middle pile begins to move down. In the excavation stage of the right tunnel, the middle pile moves up again, and the middle pile moves down again after the excavation of the right tunnel is completed. When the middle pile moves upward, the upward displacement of the pile bottom is significantly greater than that of the pile top. When the middle pile moves down, the downward displacement of the pile bottom is less than that of the pile top, which indicates that the supporting pile can play a better supporting role. The right pile has little influence on the left tunnel construction, and the deformation law of the right tunnel construction is identical to that of the left pile.

It can be observed that the supporting pile is beneficial for controlling the settlement of the station. The existing station support piles can effectively prevent the settlement of the existing station before cutting to avoid excessive subsidence.

**Figure 10:**
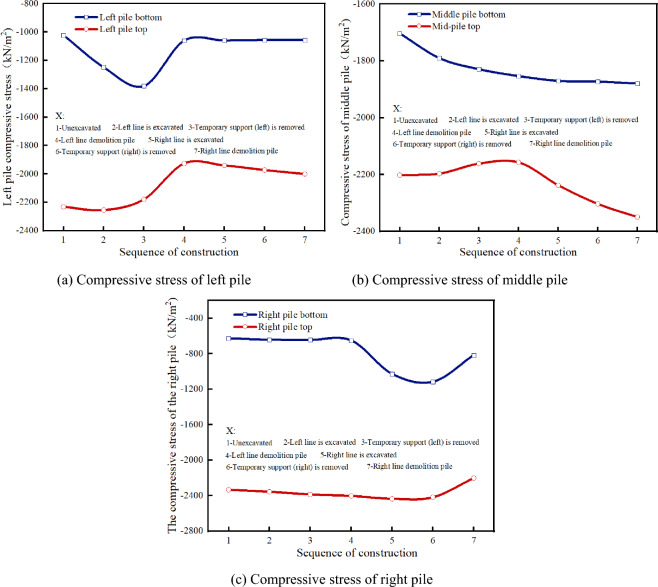
Principal stress of supporting pile.

**Figure 11:**
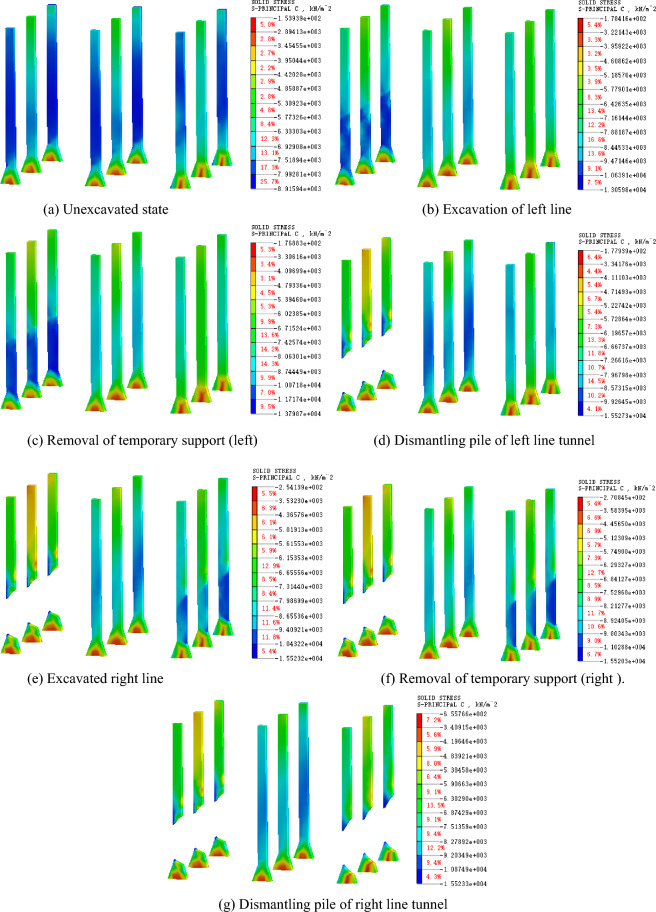
Support-pile principal-stress cloud diagram.

Figures 10 and 11 present the stress law of the existing piles at different construction stages. Before excavation, the vertical compressive stress of the left pile top is 2231.91 kN/m^2^, and that of the pile bottom is 1022.96kN/m^2^. With the excavation of the left-line tunnel, the compressive stress of the left pile increases. When the temporary support is removed, the compressive stress at the top of the left pile decreases slightly, while that at the bottom of the pile increases. The compressive stresses are 2179.81 kN/m^2^ and 1381.15 kN/m^2^, respectively. During the static pile removal, the compressive stress of the left pile decreases instantaneously. At this time, the compressive stress at the top of the pile is 1926.41 kN/m^2^, and the compressive stress at the bottom of the pile is 1061.37 kN/m^2^, which is 11.62% and 23.15% less than that before the pile removal. After the static pile removal, the compressive stress of the left pile tends to be stable.

The compressive stress of the middle pile increases continuously during the entire construction process. The compressive stress at the bottom of the middle pile increases significantly during the construction of the left tunnel and that at the top of the middle pile increases significantly during the construction of the right tunnel, which indicates that the structural load is continuously transferred to the middle pile during the entire construction process of the left and right tunnels. The right pile has little influence on the left tunnel construction, and the stress law of the right tunnel construction is consistent with that of the left pile.

**Figure 12:**
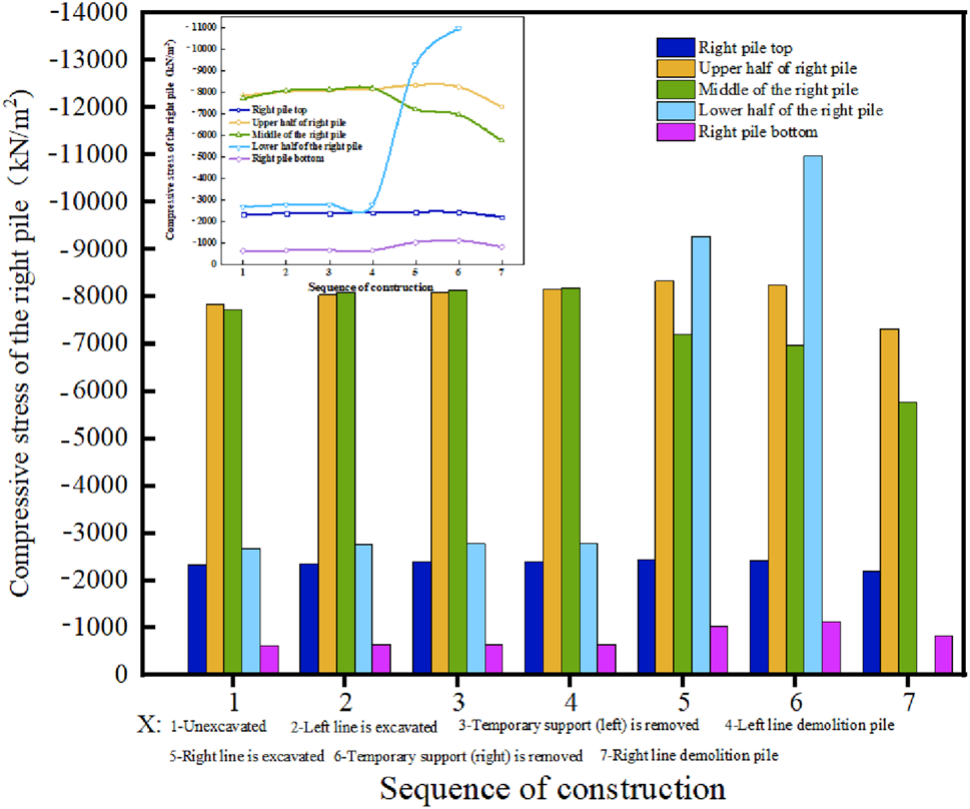
Comparison of compressive stress in different parts of right pile.

The stress variation laws of the different parts of the pile body are different, see Fig. 12. Before the excavation of the right line tunnel, the compressive stress of each part of the right pile body tends to be stable; however, the compressive stress of the upper part of the pile body is significantly greater than that of the lower part of the pile body. With the excavation of the right-line tunnel, the compressive stress of the lower half of the right pile body (i.e., the section of the right-line tunnel) increases sharply. When the right-line tunnel is excavated, the compressive stress of the lower half of the right pile body increases to 9286.33 kN/m^2^. When the temporary support is removed from the right tunnel, the compressive stress of the lower part of the right pile body increases again until the lower part of the pile body is removed, and the stress disappears. The compressive stress of the upper part of the pile tends to be stable before the pile is removed, and the compressive stress decreases after the pile is removed, but does not disappear. This is because the three linings are applied immediately after the disassembly and assembly, and the upper part of the pile can still provide support under the constraints of the three linings.

### Stress law of supporting structure of tunnel

**Figure 13:**
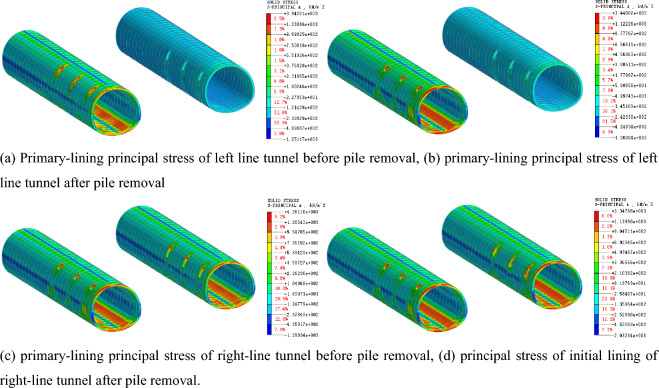
Principal-stress cloud diagram of primary lining before and after pile removal.

**Figure 14:**
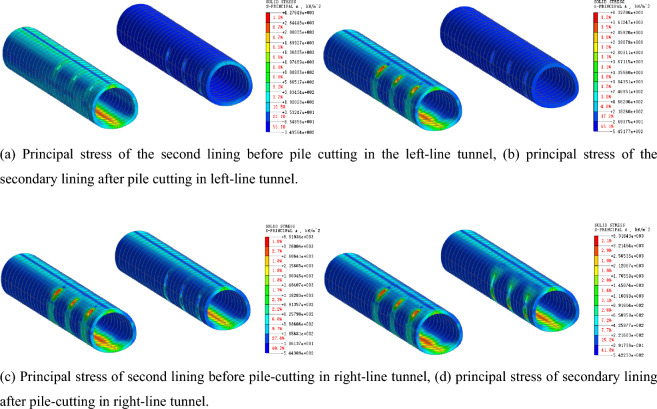
Principal-stress cloud diagram of secondary lining before and after pile removal.

After the existing piles are cut, the tunnel support structure becomes the primary bearing structure. Figures 13 and 14 present the stress characteristics of the primary support and secondary lining before and after the pile cutting, respectively. According to the analysis, before the pile cutting of the left tunnel, the maximum compressive stress of the second lining is 345.97 kN/m^2^, and the maximum tensile stress of the arch bottom is 1562.09 kN/m^2^. The maximum compressive stress of the second lining after the pile cutting is 544.85 kN/m^2^, and the maximum tensile stress at the arch bottom is 2207.32 kN/m^2^, with growth rates of 57.48% and 41.31%, respectively. Before the pile cutting of the left tunnel, the maximum compressive stress of the initial lining is 1151.17 kN/m^2^, the maximum tensile stress of the arch bottom is 1017.58 kN/m^2^, and the maximum compressive stress after the pile cutting is 1260.04 kN/m^2^. The maximum tensile stress of the arch bottom is 1126.18 kN/m^2^, and the growth rates are 9.46% and 10.67%, respectively. After the supporting pile is cut, the stress on the secondary lining increases significantly, indicating that the existing structural load s transferred from the supporting pile to the secondary lining structure. The transformation of the stress system leads to an evident settlement of the existing structure.

### Deformation law of pipe curtain

**Figure 15:**
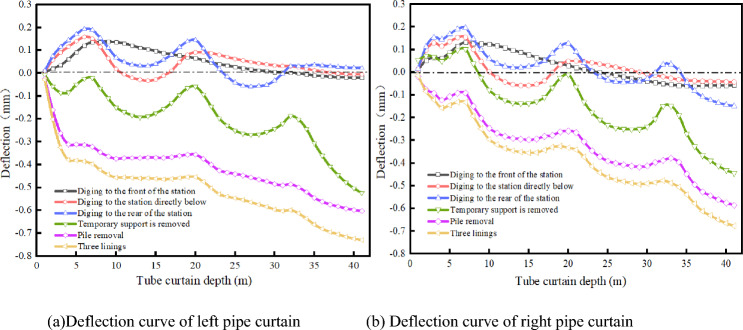
Deflection curve of pipe curtain.

As a reinforcement measure, pipe sheds have different effects on the settlement of existing stations at different construction stages. The deflection curves of the pipe curtains of the left and right two-line tunnels under different excavation footages are studied and extracted, as shown in Fig. 15. When excavated to the front of the station, the pipe roof is affected by the first supporting pile in front of the existing station, and a 0.14-mm uplift is generated near the first supporting pile. When excavation is performed directly below the station, under the influence of a second supporting pile directly below the existing station, the pipe roof produces an uplift of 0.09 mm at the position of the second supporting pile. When excavation is performed to the rear of the station, the pipe curtain is uplifted again under the influence of the third supporting pile, and the uplift value is 0.04 mm. For different excavation footages, the pipe curtain is constantly fluctuated upward under the influence of the existing pile; therefore, the pipe curtain does not play a load-bearing role in the excavation stage.

When the temporary support is removed, the pipe roof begins to settle and plays a load-bearing role. Because the supporting pile is not removed, a wave peak still exists. At this time, the mechanism of the pipe roof is similar to that of a multi-segment beam. After the static pile removal, the pipe roof undergoes a large settlement. The maximum settlement is near the supporting pile and is 0.29 mm. The wave peak disappears, the deflection curve tends to become stable, and the pipe roof mechanism is no longer a multi-segment beam. At this time, a part of the structural load is transferred from the supporting pile to the pipe roof.

## Influence of pile removal time on existing stations

### Influence of pile removal on station settlement

**Figure 16:**
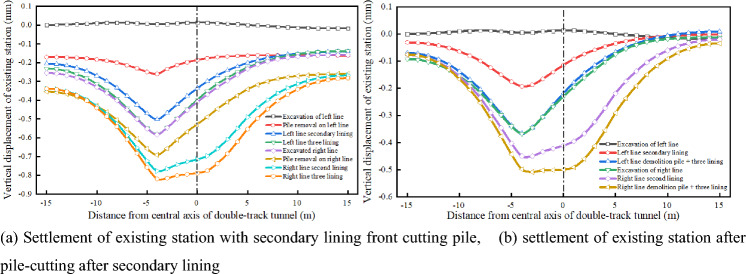
Settlement results of station at different pile-cutting times.

To study the influence of different pile-removal times on an existing station, the settlement results of the station at different pile-cutting times are obtained, as shown in Fig. 16. When the pile cutting time is varied, the final settlement of the existing station is 0.82 mm when the pile is cut before the secondary lining is applied. When the pile is cut after the secondary lining is applied, the final settlement of the existing station is 0.51 mm, and the settlement decreases by 0.31 mm. The settlement of the existing structure is 38% less than that before the secondary lining is applied. When a pile is cut before the secondary lining, the settlement of the existing station is extremely large and is difficult to control in an actual project. Therefore, cutting the piles after the secondary lining and then applying the three linings can effectively control the settlement of existing stations.

### Influence law of tunnel stress

**Figure 17:**
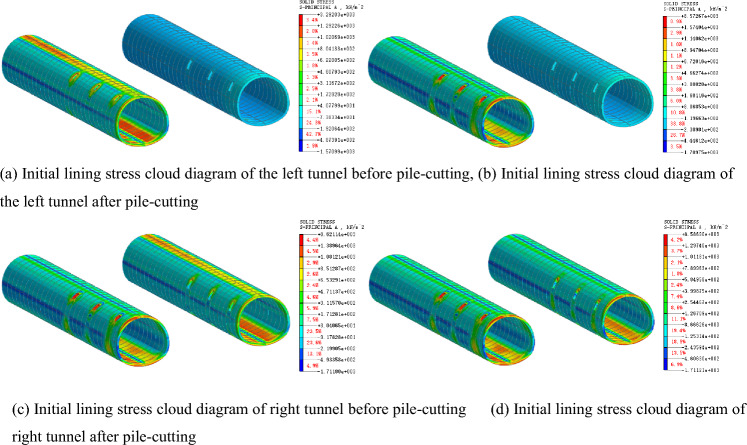
Stress cloud diagram of primary lining of pile-cutting before secondary lining.

Figure 17 presents the stress cloud diagram of the primary support of the tunnel when the pile is removed before the secondary lining. When the pile is cut before the construction of the second lining, the maximum compressive stress of the first lining before the pile-cutting of the left tunnel is 1370.99 kN/m^2^, and the maximum tensile stress at the arch bottom is 1104.12 kN/m^2^. The maximum compressive stress after the pile-cutting is 1709.75 kN/m^2^, and the maximum tensile stress at the arch bottom is 1368.98 kN/m^2^, with growth rates of 24.71% and 23.99%, respectively. The maximum compressive and tensile stress growth rates of the initial lining before and after the pile cutting are 9.46% and 10.67%, respectively. Therefore, when the timing of pile cutting is selected before the secondary lining is applied, the stress of the primary lining increases significantly after the cutting of the supporting pile. This indicates that the existing structural load is transferred from the supporting pile to the primary lining structure, and the strength of the primary lining structure is low, which is not conducive to the stability of the engineering structure.

**Figure 18:**
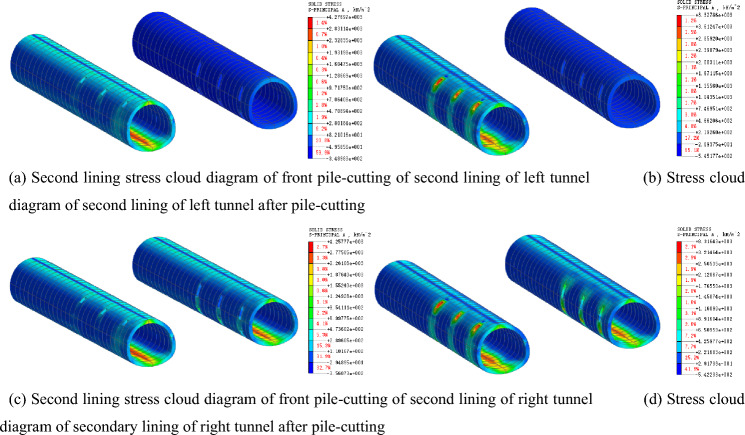
Stress cloud diagram of the second lining after pile-cutting at different times of pile-cutting.

Figure 18 presents a stress comparison of the secondary lining of the tunnel at different pile removal times. When the pile is cut before the secondary lining is applied, the maximum compressive stress on the secondary lining is 348.98 kN/m^2^, and the maximum tensile stress of the arch bottom is 1532.74 kN/m^2^. When the pile is cut after the secondary lining is applied, the maximum compressive stress of the secondary lining after the pile is cut is 544.84 kN/m^2^, and the maximum tensile stress of the arch bottom is 2207.32 kN/m^2^, which is 45.81% and 44.01% greater than those before the secondary lining, respectively. Therefore, the pile is cut after the secondary lining is applied, and the secondary lining provides full support. An increase in the stiffness of the supporting structure after the construction of the secondary lining can effectively support the existing structure.

## Field monitoring

**Figure 19:**
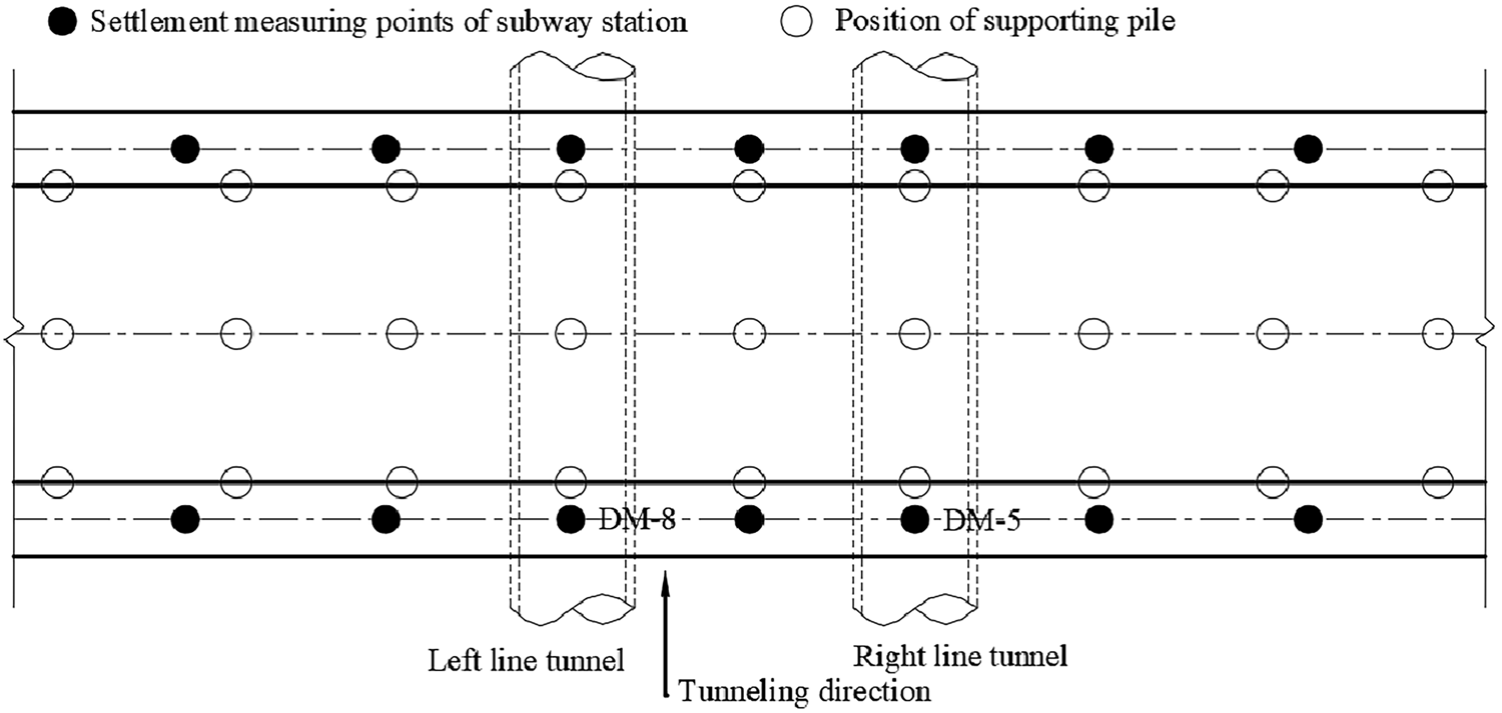
Distribution map of existing station measuring points.

The settlement of the existing station structure is monitored during the construction of the tunnel, and the measurement points are shown in Fig. 19.

**Figure 20:**
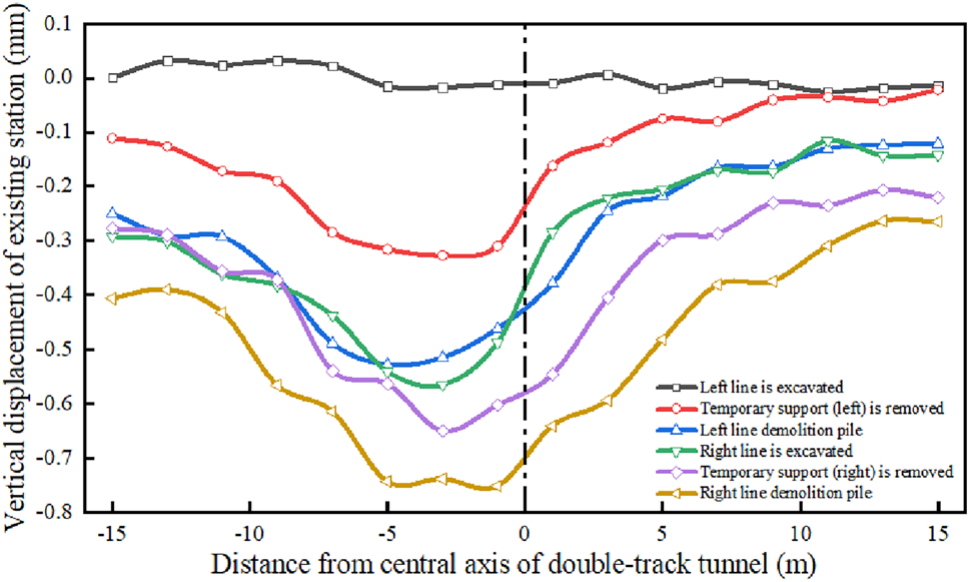
Measured station settlement at measuring points.

**Figure 21:**
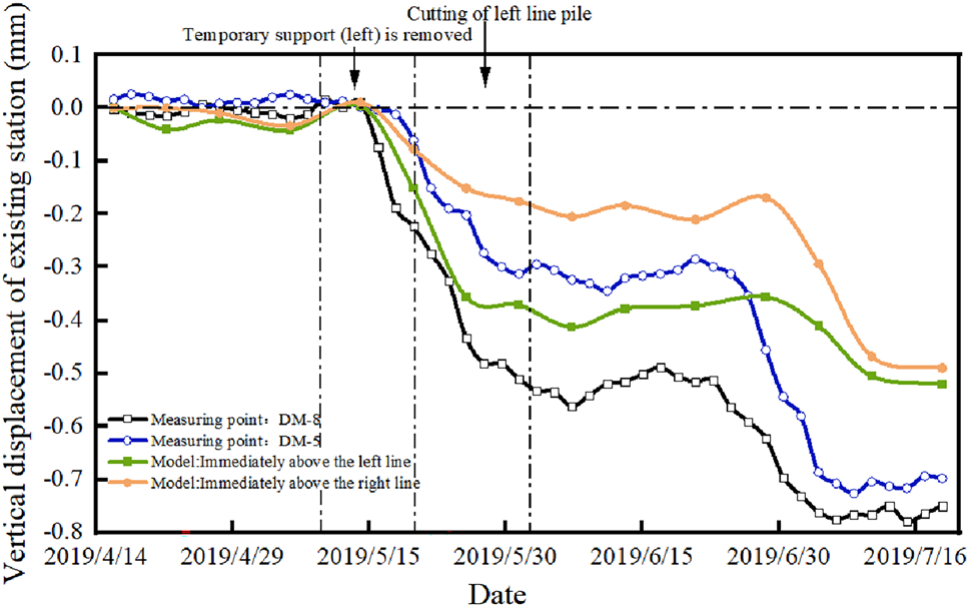
Settlement time history curve of existing station is measured at measuring points.

Figures 20 and 21 present the settlement law of the existing station measured at the measuring points during the tunnel construction. The monitoring results of the vertical displacement of the existing line structure show that the subsidence of the existing station is small before the supporting pile is cut, and the subsidence of the existing station is the greatest during the static pile removal stage. The settlement of the existing station above the tunnel is the greatest, and the settlements at both ends is small. The overall settlement curve of the existing station is consistent with the model simulation analysis results, which verifies the accuracy of the numerical simulation.

According to the on-site monitoring results, it can be observed that the maximum settlement of the existing station is 0.78 mm, and the numerical simulation results show that the maximum settlement of the existing station is 0.51 mm, which meets the specification requirements for the normal operation settlement of an existing station of 3 mm. This shows that the settlement of the existing station has been effectively controlled after the comprehensive guarantee method of surface grouting, tunnel pipe shed reinforcement + initial support strengthening + secondary lining, static demolition pile + three-lining design is adopted in the existing super-close underpass structure.

## Conclusion

1. When the tunnel passes under an existing station, the existing stations of the left and right upper excavation steps are slightly settled, and the existing stations of the left and right lower excavation steps are slightly jacked up. The settlement of the existing station is evident during the static pile removal stage and the removal of the temporary support. The settlement in the static pile removal stage is the greatest and accounts for 60.79% of the total settlement of the existing station, which is the control stage of the construction.
2. The supporting pile of the existing station has a better supporting effect on the existing station before cutting. After cutting, the upper half of the pile body can still play a supporting role under the constraints of the three linings, which inhibits the subsidence of the subway station. After cutting the supporting pile, the stress on the lining of the underpass tunnel increases significantly, indicating that the supporting effect of the existing station is transferred from the existing pile to the new tunnel structure. The change in the stress system caused by the cutting piles results in a significant increase in the settlement of existing stations.
3. The pipe curtain near the supporting pile is uplifted during the tunnel excavation stage, indicating that the pipe curtain does not play a supporting role in the existing station before the pile cutting; the settlement of the pipe curtain is observed after the pile-cutting, thus indicating that the load of the existing station is transferred from the supporting pile to the tunnel structure and the pipe curtain after pile cutting, which demonstrates the supporting effect of the pipe curtain.
4. The cutting time of the existing pile significantly influences the deformation of the existing station. When a secondary lining is used to cut the pile before the construction, the load of the existing station is borne by the primary support, and the stiffness of the primary support is low. In this regard, the existing pile should be cut after the construction of the secondary lining, and the stiffness of the supporting structure after the secondary lining construction is significantly increased. The settlement of the existing station is reduced by 38% compared with that before secondary lining construction.
5. The field investigation shows that the reinforcement measures of surface grouting + pipe shed + multilayer lining ensures the stability of the force system after pile-cutting and effectively restrains the deformation of the existing station, and the settlement of the station is less than 1 mm.

## Data Availability

Some or all data, models, or code that support the findings of this study are available from the corresponding author upon reasonable request.
